# ZNF804a Regulates Expression of the Schizophrenia-Associated Genes PRSS16, COMT, PDE4B, and DRD2

**DOI:** 10.1371/journal.pone.0032404

**Published:** 2012-02-27

**Authors:** Matthew J. Girgenti, Joseph J. LoTurco, Brady J. Maher

**Affiliations:** 1 Department of Physiology and Neurobiology, University of Connecticut, Storrs, Connecticut, United States of America; 2 Lieber Institute for Brain Development, Johns Hopkins Medical Campus, Baltimore, Maryland, United States of America; University College London, United Kingdom

## Abstract

ZNF804a was identified by a genome-wide association study (GWAS) in which a single nucleotide polymorphism (SNP rs1344706) in ZNF804a reached genome-wide statistical significance for association with a combined diagnosis of schizophrenia (SZ) and bipolar disorder. Although the molecular function of ZNF804a is unknown, the amino acid sequence is predicted to contain a C2H2-type zinc-finger domain and suggests ZNF804a plays a role in DNA binding and transcription. Here, we confirm that ZNF804a directly contributes to transcriptional control by regulating the expression of several SZ associated genes and directly interacts with chromatin proximal to the promoter regions of PRSS16 and COMT, the two genes we find upregulated by ZNF804a. Using immunochemistry we establish that ZNF804a is localized to the nucleus of rat neural progenitor cells in culture and in vivo. We demonstrate that expression of ZNF804a results in a significant increase in transcript levels of PRSS16 and COMT, relative to GFP transfected controls, and a statistically significant decrease in transcript levels of PDE4B and DRD2. Furthermore, we show using chromatin immunoprecipitation assays (ChIP) that both epitope-tagged and endogenous ZNF804a directly interacts with the promoter regions of PRSS16 and COMT, suggesting a direct upregulation of transcription by ZNF804a on the expression of these genes. These results are the first to confirm that ZNF804a regulates transcription levels of four SZ associated genes, and binds to chromatin proximal to promoters of two SZ genes. These results suggest a model where ZNF804a may modulate a transcriptional network of SZ associated genes.

## Introduction

Schizophrenia (SZ) is a heritable disorder having no single gene or environmental factor that accounts for a majority of cases. An emerging hypothesis to explain SZ etiology suggests that epistatic interactions between multiple susceptibility genes all converge onto a set of biological networks important for SZ pathology [1], [2]. If SZ associated genes are closely connected to a common disease pathway, then controlling the transcription of genes within this network would be critical for proper biological function. Therefore, we hypothesize that transcription factors associated with SZ may be central to a transcriptional network that regulates the expression of other SZ associated genes.

Genome-wide association studies (GWAS) are a powerful method to identify single nucleotide polymorphisms (SNPs) that are associated with a diagnosis. The first gene to reach genome-wide significance for psychosis was ZNF804a when SNP rs1344706 was significantly associated when schizophrenia and bipolar disorder diagnoses are combined [3]. Several follow up GWAS studies have replicated the association of rs1344706 with SZ in different populations [4], [5], [6], and several other SNPs near the ZNF804a locus have a significant association with SZ [7], [8].

In many cases, SNPs significantly associated with a particular diagnosis are located within intronic regions and therefore do not alter the coding sequence of proteins, but rather may affect the binding of transcription factors or alter the post-transcriptional splicing of nearby genes. The rs1344706 SNP has been correlated with increased ZNF804a transcript levels in adult tissue [4], [9], and the risk allele has a decreased affinity for nuclear proteins compared to the common allele [10]. RNAi knockdown of ZNF804a in an immortalized human neuroepithelium cell line showed altered expression of genes involved in cell adhesion [11]. Although ZNF804a is a relatively strong candidate susceptibility gene, the function of the protein and the molecular mechanism responsible for enhancing risk for psychosis remains unknown. Our overall aim was to determine the function of ZNF804a by testing its ability to alter transcription of other SZ associated genes and by performing chromatin immunoprecipitation assays (ChIP) to determine if ZNF804a is specifically associated with promoter regions of the regulated genes we identified. To this end, we identify four SZ associated genes whose transcripts are regulated by the expression level of ZNF804a in rat cortical progenitor cells. In addition, we show that ZNF804a is associated with the promoters of the two up-regulated genes in our study.

## Results

**Figure 1:**
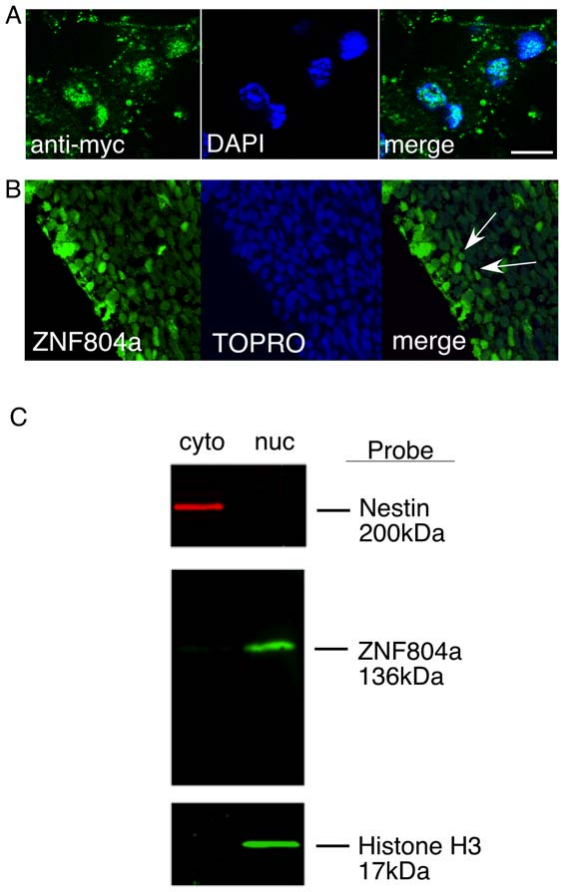
Recombinant and endogenous ZNF804a is localized to the nucleus of neural progenitor cells. **A**) pCAG-ZNF804A was transfected into rat neural progenitor cells for twenty-four hours and processed for immunocytochemistry with antibodies against an myc-tag fused to ZNF804a. Expression of ZNF804a co-localizes with the nuclear counter stain DAPI (Scale bar = 10 µm). **B**) Immunohistochemistry using anti-ZNF804a antibodies showing endogenous ZNF804a protein (left panel) co-localizes (right panel) with the nuclear counter stain TOPRO (middle panel) in E11 neural progenitor cells within the ventricle zone. **C**) Endogenous ZNF804a protein is devoid of the cytoplasmic fraction and observed in the nuclear fraction. As a control to demonstrate proper cellular fractionation, Nestin, a cytoplasmic marker of neural progenitor cells, is observed in the cytoplasmic fraction. Likewise, Histone H3, a chromatin marker, is observed in the nuclear fraction.

SZ is a neurodevelopmental disorder with symptoms associated with defects in cortical circuitry [12]. Therefore, we assayed transcript levels in neural progenitors isolated from rat forebrain at embryonic day 11 (E11). This population of progenitor cells give rise to cortical and striatal neurons during embryonic development. During this developmental time window, this population of progenitor cells express ZNF804a transcript as measured by qRT-PCR (data not shown). To identify SZ associated transcripts that are regulated downstream of ZNF804a, we subcloned human ZNF804a into a plasmid with a constitutive promoter (pCAG) creating pCAG-hZNF804a. We also added an epitope-tag to ZNF804a so we could localize the expressed protein and perform ChIP assays. 24 hours after transfection of pCAG-hZNF804a immunocytochemistry with anti-myc-tag antibodies revealed a predominantly nuclear localization of ZNF804a (Figure 1A). We also observed nuclear localization of endogenous ZNF804a protein in cortical progenitor cells in vivo using an antibody directed against ZNF804a protein (Figure 1B). Furthermore, using subcellular fractionation, we observed endogenous ZNF804a protein was absent in cytoplasmic fraction but was clearly present in the nuclear fraction (Figure 1C). These results suggest endogenous ZNF804a is localized in the nucleus of neural progenitor cells both in vitro and in vivo, and our recombinant ZNF804a expression construct has a similar localization pattern to that observed for endogenous ZNF804a protein.

**Figure 2:**
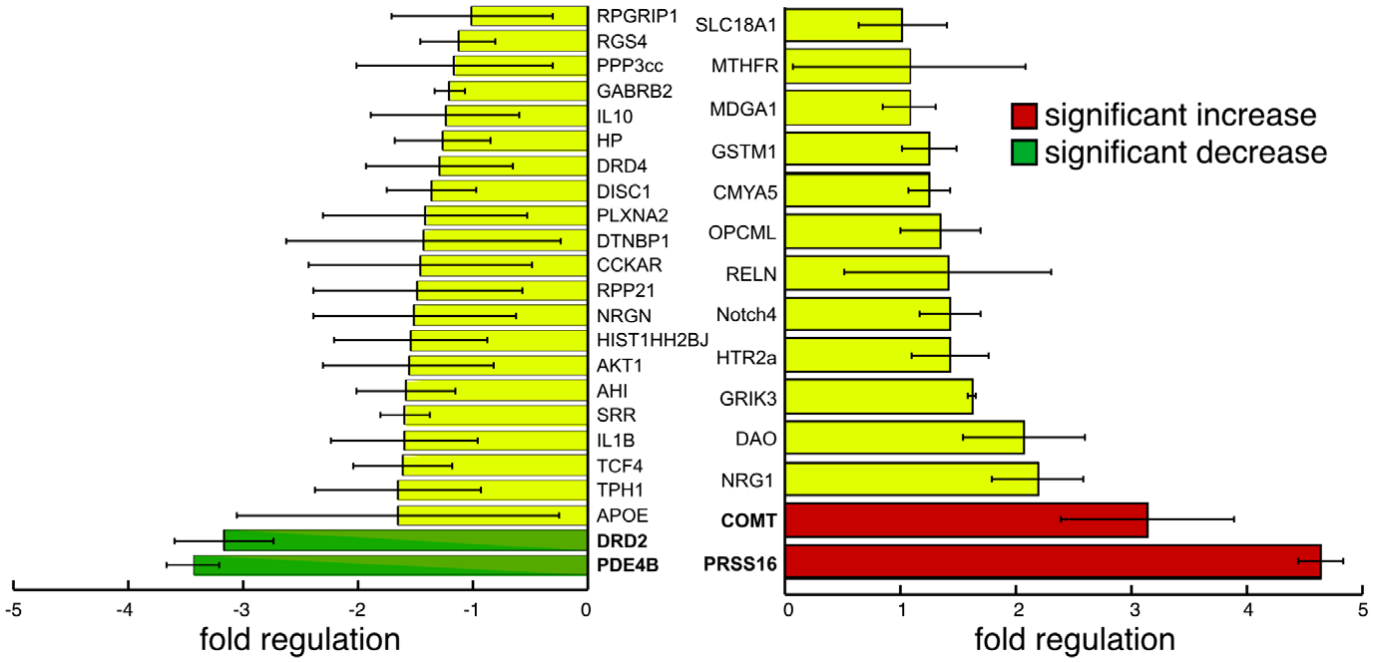
ZNF804a regulates the transcription of several Schizophrenia- associated genes. **A**) Quantitative RT-PCR was performed on 37 SZ associated gene transcripts following ZNF804A transfection. PRSS16 and COMT (red) showed robust upregulation of transcription twenty-four hours after transfection (Bonferroni corrected p<0.05; n = 5). Conversely, PDE4B and DRD2 (green) transcript levels were downregulated following ZNF804a transfection (Bonferroni corrected p<0.05; n = 5).

We next asked if overexpression of ZNF804a in our neural progenitor cell cultures could alter the transcript levels of other SZ associated genes. We designed primers for the top 37 SZ associated genes highlighted on the SZgene.org website (Table S1; www.szgene.org/TopResults.asp; as of May 2011) [13] and assayed mRNA levels by qRT-PCR. Neural progenitor cell cultures where transfected for 24 hours with either pCAG-ZNF804a or pCAG-GFP (transfection control) followed by isolation of RNA. We observed a statistically significant increase in transcript levels for two genes (Figure 2, red; COMT 3.14±0.75 and PRSS16 4.64±0.2; Bonferroni corrected p<0.05; n = 5) and a statistically significant decrease in transcript levels for two genes (Figure 2, green; PDE4B −3.43±0.23 and DRD2 −3.17±0.43; Bonferroni corrected p<0.05; n = 5) relative to GFP transfected controls. These results indicate that ZNF804a expression can modulate gene transcription, however with these results it is unclear whether ZNF804a expression is directly and/or indirectly regulating transcription.

**Figure 3:**
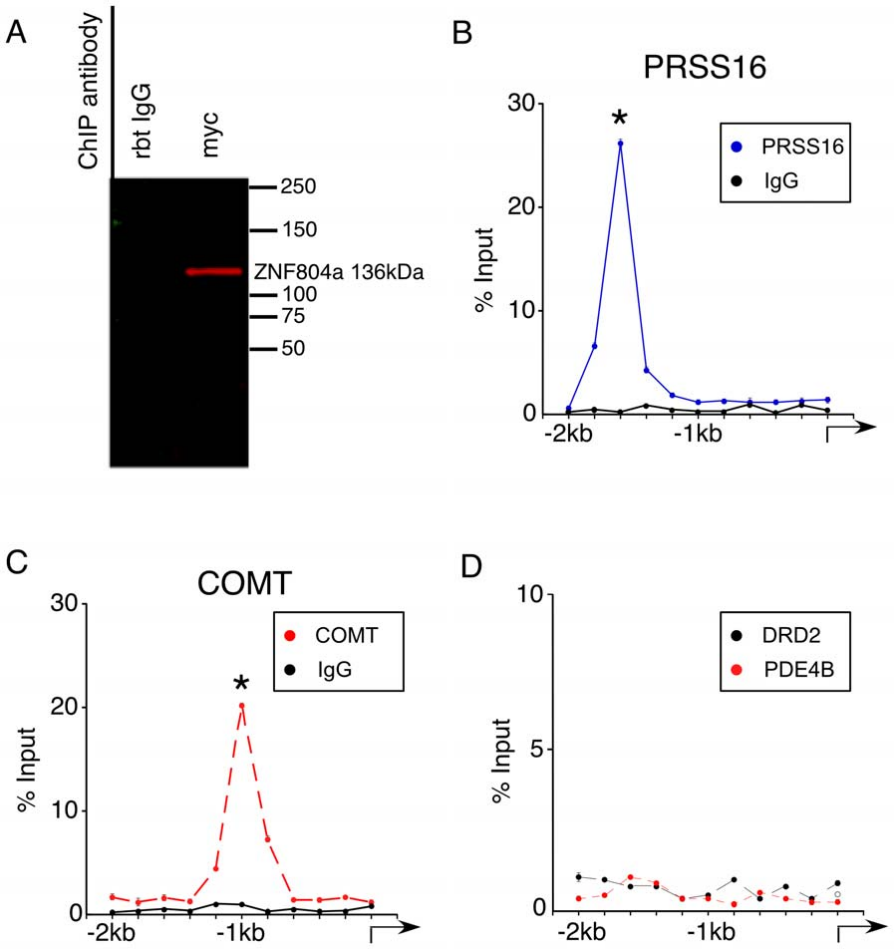
Recombinant ZNF804a binds to the DNA regions directly upstream of the transcription start site of PRSS16 and COMT. **A**) Chromatin was immunoprecipitated with antibody directed against the myc-tag or IgG as control and then probed with anti-ZNF804a antibody. Recombinant ZNF804a was correctly identified by anti-ZNF804a antibody. **B–C**) Tiling qRT-PCR of the promoter sequences of PRSS16 and COMT following ChIP against the myc-tag reveals enrichment for ZNF804A. Enrichment of ZNF804A on the PRSS16 promoter appears 1.5 Kb 5′ upstream of the transcription start sites (TSS) while enrichment on the COMT promoter appears 1 Kb 5′ upstream of the TSS **D**) Tiling qRT-PCR of the promoter sequences of DRD2 and PDE4B following ChiP did not result in enrichment of ZNF804a (all figures *p<0.05, *Students* t-test, Error bars indicate ± SEM).

To examine if ZNF804a expression may directly regulate transcription, we performed ChIP to determine whether ZNF804a directly interacts with genomic regions proximal to the promoters of COMT, PRSS16, PDE4B, and DRD2, the four regulated genes indentified in our qRT-PCR assays. Chromatin samples were isolated from progenitor cultures transfected with pCAG-ZNF804a. Proteins were cross-linked to DNA prior to isolation and then sheered, chromatin was immunoprecipitated with an anti-myc antibody, crosslinks reversed, and isolated DNA subjected to qRT-PCR with a set of tiled primers spanning 2 kb upstream of the annotated transcription start sites for PRSS16, COMT, PDE4B, and DRD2 that included a GC-rich region (Table S2; PRSS16, chr17:50145344-501473440, COMT ch11:84579705-84581705; PDE4B, chr5:133600726-133602726 and DRD2, chr8: 50582050-50584050). To confirm our epitope-tagged expression construct, we immunprecipitated protein from transfected cells with anti-myc-tag antibody and detected it in western blot with an anti-ZNF804a antibody (136 kDa; Figure 3a). For our ChIP assays 1 µg of DNA was immunoprecipitated with anti-myc-tag antibody or negative control IgG and expressed as a percentage of input. Chromatin immunoprecipitated for ZNF804a-myc was enriched in single distinct peaks within the PRSS16 (Figure 3b; 26.2±0.4%, p<0.05; n = 4) and COMT (Figure 3c; 20.2±0.2%, p<0.05; n = 4) 2 kB regions, but not within 2 kB upstream of PDE4B or DRD2 genes (Figure 3d). Furthermore, the genomic fragments that immunoprecipitated with ZNF804a contained predicted zinc-finger interacting motifs (COMT GGCGG; PRSS16 GGCG). These results indicate that overexpressed ZNF804a directly interacts with the promoter regions of PRSS16 and COMT and is consistent with ZNF804a having a direct positive effect on the transcription of these two SZ associated genes.

**Figure 4:**
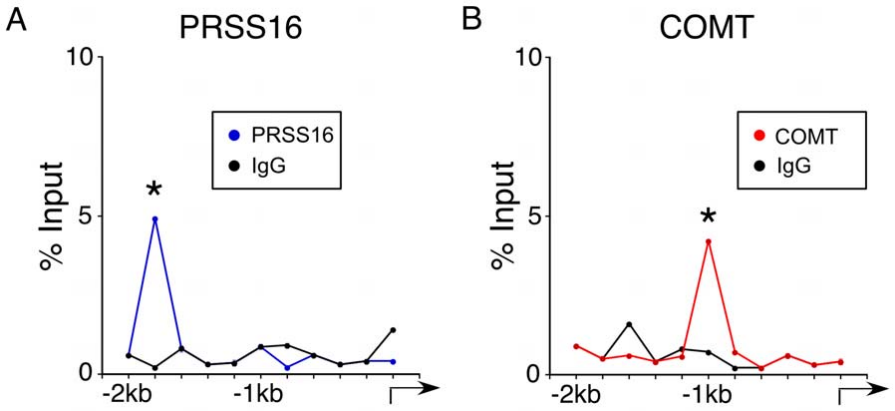
Endogenous ZNF804a binds to the DNA regions directly upstream of the transcription start site of PRSS16 and COMT. **A**) Tiling qRT-PCR of the promoter sequences of PRSS16 and COMT following ChIP using anti-ZNF804a reveals enrichment for ZNF804A. Enrichment of ZNF804A on the PRSS16 promoter appears 1.5 Kb 5′ upstream of the transcription start sites (TSS) while enrichment on the COMT promoter appears 1 Kb 5′ upstream of the TSS.

To rule out the possibility that overexpression of ZNF804a results in artificial binding to DNA upstream for PRSS16 and COMT, we determined whether endogenous ZNF804a protein interacts with the same regions of PRSS16 and COMT. We performed ChIP assays using anti-ZNF804a antibody in untransfected neural progenitor cell cultures. Similar to ChIP results for the overexpressed ZNF804a protein ChIP for endogenous ZNF804a protein enriched DNA sequence within the PRSS16 (Figure 4a; 4.9±0.03%, p<0.05; n = 5) and COMT (Figure 4b; 4.2±0.06%, p<0.05; n = 5) promoter regions. Moreover, the enriched peaks were in the same locations for recombinant ZNF804a and endogenous ZNF804a. These results are consistent with ZNF804a contributing directly to the regulation of transcription.

## Discussion

This study identifies several putative SZ-associated genes that are transcriptionally regulated by ZNF804a overexpression. We show that endogenous and recombinant ZNF804a localize to the nucleus of neural progenitor cells in vitro and in vivo. In addition, we show a direct interaction of endogenous and recombinant protein interacting with regions immediately upstream of the start sites of PRSS16 and COMT, two genes we observed that were upregulated by ZNF804a expression.

Three genes we observed to be regulated by ZNF804a expression are directly involved in dopaminergic transmission and cAMP signaling, two pathways thought to underlie many of the symptoms of psychosis. Specifically, COMT is an enzyme that degrades catecholamines in the synaptic cleft and SNPs within the coding sequence of this gene are associated with cognitive deficits [14]. DRD2 encodes the D2 subtype of the dopamine receptor and activation of this receptor by dopamine inhibits adenylate cyclase activity. PDE4B encodes the cAMP-specific phosphodiesterase 4B, an enzyme responsible for the breakdown of cAMP. We find this grouping of regulated genes to be very interesting because they are closely associated with a common signaling pathway. These genes are clearly important for adult brain function, but determining how altered prenatal expression of these genes will effect neurodevelopment will need further investigation.

Direct binding of ZNF804a to its promoter region also positively regulated the expression level of PRSS16. PRSS16 encodes a serine protease located on chromosome 6 near the major histocompatability complex (MHC) class I region of the genome. A SNP in this region of the genome (6p22.1) reached genome-wide association for SZ [15], [16]. Expression of this gene is most abundantly observed in the thymus, however expression is found in other tissues including brain [17]. PRSS16 is an interesting gene because of its role in immune function. Immune function is thought to be important in the etiology of SZ as prenatal influenza increases risk of SZ [18]. However, the function of PRSS16 during brain development is unknown.

Given the intronic location of SNP rs1233706 within the ZNF804a gene, altered expression or splice variation may confer SZ susceptibility. The alleles of SNP rs1344706 are shown to differentially bind nuclear proteins and suggest a molecular mechanism for altering ZNF804a expression [10]. In addition, the rs1344706 risk allele is associated with increased expression in the dorsolateral prefrontal cortex [4]. However, in lymphoblastoid cells, increased expression generally associates with SNP rs1344706, however rs1344706 does not appear to be the only cis-acting eQTL responsible for expression [9]. It will be important in future studies to better characterize how this SNP alters expression in fetal and adult brain to more precisely match appropriate expression levels in cell and animal models.

Beyond developmental expression, understanding the function and determining the genes that are regulated by ZNF804a either directly through promoter/enhancer occupancy or indirectly through downstream molecular pathways is relevant to our understanding of the function of this gene and how it may associate with SZ. Regardless of the expression pattern observed in SZ, our data and others suggest that transcription downstream of ZNF804a is sensitive to the expression level of ZNF804a [11]. Using a whole-genome microarray approach, Hill et al. [11] showed RNAi knockdown of ZNF804a in human neural progenitor cell line resulted in altered expression of 151 unique genes, however this study could not ascertain if knockdown of ZNF804a produced a direct or indirect effect on gene expression. Our ChIP results expand our understanding of ZNF804a function by providing evidence of ZNF804a directly regulating transcription through promoter/enhancer occupancy. It will be useful in future experiments to expand these results by using a genome-wide ChIP sequencing approach. Collectively, these results strongly suggest ZNF804a expression level is a candidate mechanism for conferring risk.

## Materials and Methods

### Ethics Statement

All studies were conducted in accordance with protocols that were approved by the University of Connecticut Institutional Animal Care and Use Committee (IACUC; Assurance No. A09-025, 2/2011). The facilities at the University of Connecticut are accredited by the Association for the Assessment and Accreditation of Laboratory Animal Care (AAALAC).

### Nuclear isolation

Nuclei were extracted from confluent neural progenitors (∼90%) using the CelLytic Nuclear extraction Kit from Sigma (NXTRACT). Briefly, cells were swelled with a hypotonic buffer. They were then disrupted and cytoplasmic extract was removed. The nuclear extract was isolated from the remaining lysate with a high salt buffer.

### Western Blot Analysis

Chromatin recovered by myc-tag or IgG immunoprecipitation from rat neural progenitors was resolved by 15% sodium dodecyl sulfate polyacrylamide gel electrophoresis (SDS-PAGE), transferred to nitrocellulose, probed with a ZNF804a antibody (Santa Cruz Biotechnology), and visualized on an Odyssey Infrared Imager.

### Immunohistochemistry

Brains were dissected from E13 rat embryos. Paraffin embedded brains were cut on a cryostat. Cortical sections were taken and immunohistochemistry was performed. Sections were stained using antibodies against ZNF804A (1∶500; Santa Cruz Biotechnology) with a nuclear counter stain TO-PRO (Invitrogen, T-3605). Anti-rabbit 488 secondary antibody (1∶400, Molecular Probes) was used for fluorescent imaging. Images were taken with a Leica TCS SP2 confocal microscope.

### Quantitative PCR

RNA (1 µg) was reverse-transcribed into cDNA using oligo-dT primers and reverse-transcriptase. RNA was then hydrolyzed, and re-suspended in nuclease-free water. Gene-specific primers were designed using Primer 3 software (http://frodo.wi.mit.edu/primer3/) and tested for efficiency and specificity by serial dilutions and melt curve analysis. Sybr Green mix (ABI) was used to amplify cDNA. Primer sequences are in Table S1.

### Chromatin Immunoprecipitation

Chromatin immunoprecipitations were performed as described with several modifications (below). Briefly, chromatin isolated from rat neural progenitors (10^6^) was sonicated into 200 to 500 base pair (bp) fragments. Resulting samples were immunoprecipitated with 5 µg myc-tag antibody (Abcam, ab9132), 10 µg ZNF804a antibody (Santa Cruz Biotechnology), or 5 µg normal rabbit immunoglobulin G (IgG) (Sigma I8140). Immunoprecipitated DNA was probed by qRT-PCR using promoter specific tiling primers across the promoters of PRSS16, COMT, DRD2 and PDE4B. Primer sequences are in Table S2.

## Supporting Information

Table S1 Table of all the gene names and primers used in qRT-PCR experiments. Threshold values for qPCR in each condition, wild type (WT) and wild type transfected with ZNF804A (ZNF) followed by the first delta Ct after Gapdh normalization. Fold Regulation was calculated and a standard error of the mean for each condition.
(TIF)

Table S2 Tiling primers used in ChIP experiments. Table of tiling primers of promoters for qRT-PCR combined with chromatin immunoprecipitation. All primers are ordered by distance from the transcription start site (TSS) of each gene starting with 1 (2 Kb away) to 8 (on the TSS).
(TIF)
