# Supplementary figures and images for: ZNF804a Regulates Expression of the Schizophrenia-Associated Genes PRSS16, COMT, PDE4B, and DRD2
Source: PLoS One. 2012 Feb 27;7(2):e32404. doi: 10.1371/journal.pone.0032404 (PMC3288100; doi:10.1371/journal.pone.0032404)

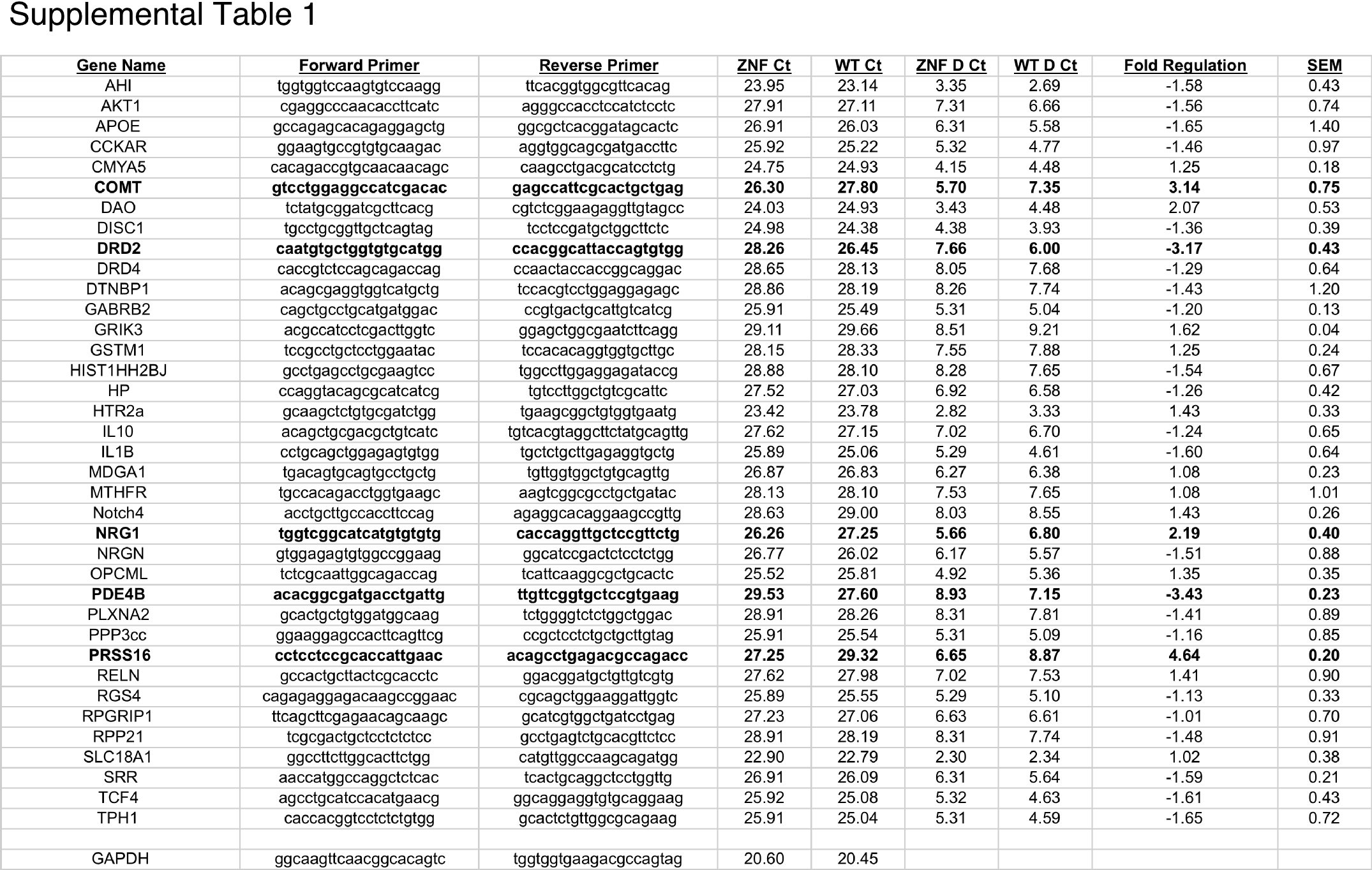

Supplement: Table S1 — Table of all the gene names and primers used in qRT-PCR experiments. Threshold values for qPCR in each condition, wild type (WT) and wild type transfected with ZNF804A (ZNF) followed by the first delta Ct after Gapdh normalization. Fold Regulation was calculated and a standard error of the mean for each condition. (TIF) [file pone.0032404.s001.tif]

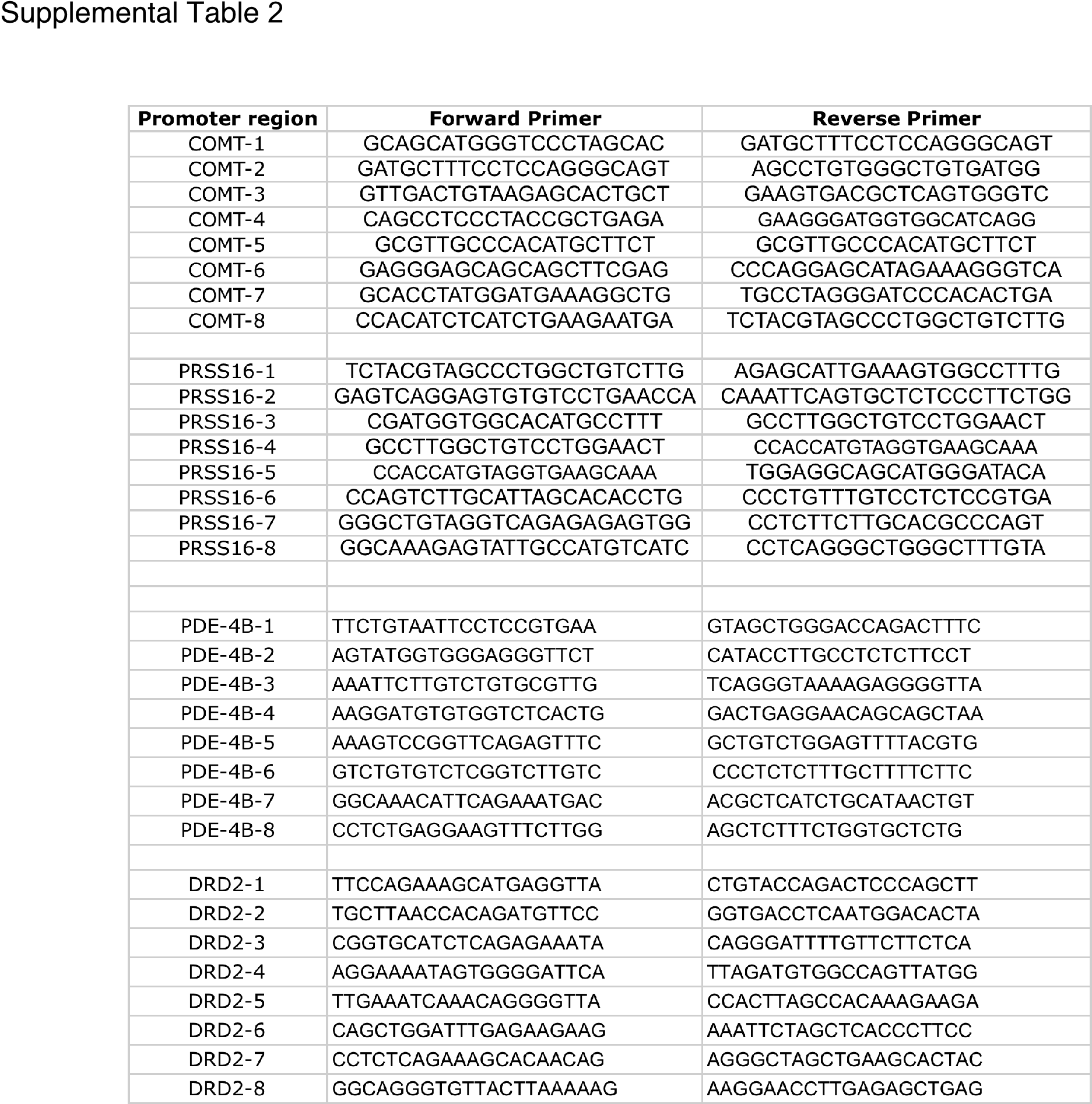

Supplement: Table S2 — Tiling primers used in ChIP experiments. Table of tiling primers of promoters for qRT-PCR combined with chromatin immunoprecipitation. All primers are ordered by distance from the transcription start site (TSS) of each gene starting with 1 (2 Kb away) to 8 (on the TSS). (TIF) [file pone.0032404.s002.tif]
